# Initial CT Imaging Predicts Mortality in Severe Traumatic Brain Injuries in Pediatric Population—A Systematic Review and Meta-Analysis

**DOI:** 10.3390/tomography9020044

**Published:** 2023-02-27

**Authors:** Doris Goubran, Divjeet Batoo, Janice Linton, Jai Shankar

**Affiliations:** 1 Department of Radiology, Rady Faculty of Health Sciences, University of Manitoba, Winnipeg, MB R3A 1R9, Canada; 2 Indigenous Health Liaison Librarian, Neil John Maclean Health Sciences Library, Winnipeg, MB R3E 3P5, Canada; 3 Department of Human Anatomy and Cell Science, Rady Faculty of Health Sciences, University Of Manitoba, Winnipeg, MB R3E 0W2, Canada; 4 Biomedical Engineering, Price Faculty of Engineering, University of Manitoba, Winnipeg, MB R3T 5V6, Canada

**Keywords:** CT imaging, mortality, pediatric, traumatic brain injuries

## Abstract

The purpose of this systematic review was to analyze evidence based on existing studies on the ability of initial CT imaging to predict mortality in severe traumatic brain injuries (TBIs) in pediatric patients. An experienced librarian searched for all existing studies based on the inclusion and exclusion criteria. The studies were screened by two blinded reviewers. Of the 3277 studies included in the search, data on prevalence of imaging findings and mortality rate could only be extracted from 22 studies. A few of those studies had patient-specific data relating specific imaging findings to outcome, allowing the data analysis, calculation of the area under the curve (AUC) and receiver operating characteristic (ROC), and generation of a forest plot for each finding. The data were extracted to calculate the sensitivity (SN), specificity (SP), positive predictive value (PPV), negative predicted value (NPV), AUC, and ROC for extradural hematoma (EDH), subdural hematoma (SDH), traumatic subarachnoid hemorrhage (tSAH), skull fractures, and edema. There were a total of 2219 patients, 747 females and 1461 males. Of the total, 564 patients died and 1651 survived; 293 patients had SDH, 76 had EDH, 347 had tSAH, 244 had skull fractures, and 416 had edema. The studies included had high bias and lower grade of evidence. Out of the different CT scan findings, brain edema had the highest SN, PPV, NPV, and AUC. EDH had the highest SP to predict in-hospital mortality.

## 1. Introduction

Traumatic brain injuries (TBIs) are tragic but relevant health concerns for health care professionals around the globe, as they are known to deteriorate the quality of life of healthy individuals [1]. Common causes of TBIs for children include accidental falls and motor vehicle crashes [2]. Accidental falls tend to happen with high-risk behaviors in children, especially young boys. Countries such as the United States, who have a greater availability of guns, tend to have more pediatric TBIs related to gun violence in comparison to countries that have much lower gun use [2]. Child abuse and/or shaken baby can also cause head injuries and severe TBIs [2,3]. TBIs may be closed or open head injuries, and they may be accidental or inflicted trauma. TBIs are categorized based on severity as defined by the Glasgow Coma Score (GCS) (see Table 1). The GCS measures ocular, verbal, and motor responses of head-injured patients. A score of 15 suggests normal functioning while a score of 3 indicates that the patient is unresponsive. Patients with severe TBIs have a GCS ≤ 8, while patients with a GCS > 8 are categorized as moderate (9–12) or mild (13–15).

Management of severe TBIs in children is resource-intensive, with high morbidity and poor prognosis [2,3,4]. The outcome for a severe TBI can be fatal or result in permanent severe to moderate disability. However, with correct management and immediate treatment, the outcome of a severe TBI may be positive, and patients may return to normal life with little to no adjustment. The initial prognosis of severe pediatric TBIs is essential in order to deliver the appropriate treatment, determine the intensity of treatment, and optimize different departments and resources, all while working efficiently to deliver the best quality of care [5,6,7]. However, initial prognostication of severe TBIs in children is difficult, as it requires interdisciplinary coordination and careful resource management. This is particularly important for areas that have limited resources, such as rural municipalities. Many underprivileged areas may not have any of the prognostication tools, such as CT, necessary to provide an accurate clinical picture on admission. These patients may have to be transported to larger urban centers, and the delay in receiving an initial CT could lead to a delay in much-needed treatment. Health outcomes are intersectional—the outcome of severe TBIs not only depends on the initial clinical picture, but also the availability of resources and prognostication tools. Many studies have identified a number of initial clinical and imaging findings that are useful for prognosis; nonetheless, inconsistencies about prognostication factors for severe TBIs in children exist between studies. Hence, this systematic review seeks to answer the following question: can imaging upon admission aid in prognosis of pediatric severe TBI? We hypothesize that the initial imaging data are a valuable tool in the prognosis of severe pediatric TBI. The purpose of this systematic review is to identify the imaging findings that have predictive value for outcomes to make informed decisions about resource management. The results of this study could be used to demonstrate the importance of CT and initial clinical picture and explain why these resources should be available in more places.

## 2. Materials and Methods

### 2.1. Literature Search

A librarian (J.L.) with experience in systematic reviews created a search strategy to identify studies on predicting the morbidity, mortality, and other outcomes in TBIs using early CT. The Cochrane Library of reviews and Scopus databases were searched to see if similar reviews had been conducted, and none were found. Database searches to find studies on this topic were conducted in Medline and Embase using OVID, with no date limitations applied. Searches were carried out on 3 July 2020. Details of the searches including subject headings, keywords and filters used can be found in Supplementary File S1.

### 2.2. Inclusion and Exclusion Criteria

Table 1 is a summary of the inclusion and exclusion criteria. The Glasgow Outcome Scale (GOS) mentioned in the inclusion criteria is a measure of patient recovery. A GOS of 5 indicates that the patient had good recovery, a score of 4 means moderate disability, a score of 3 is severe disability, a score of 2 represents an outcome of a vegetative state, and a GOS of 1 indicates death. For this systematic review, the scores were divided into two categories: a GOS of 1 indicated death while a GOS of 2–5 indicated survival. The rationale for this decision was to maximize the data for the meta-analysis. Although all the studies included in this systematic review used GOS (hence fulfilling the inclusion criteria), the studies had different ways of reporting the GOS. For example, some studies grouped patients into GOS 1, GOS 2–3, and GOS 4–5, while other studies reported GOS 1 to 5 separately. However, GOS 1 (death) was always separate from the other outcomes, making it possible for the authors of this systematic review to perform the meta-analysis.

The search was loaded in Covidence and duplicates were eliminated. First, the abstracts and titles were screened by two independent reviewers (D.G. and D.B.). Then, the full text was also reviewed independently by each reviewer. Studies that met all the inclusion criteria and did not meet any exclusion criteria were kept. Studies that did not meet the inclusion criteria and/or met exclusion criteria were excluded. Any conflict was resolved by consensus discussion between the reviewers. If consensus could not be reached by the two reviewers, a third individual (J.S.) made the final decision.

### 2.3. Data Extraction

The two reviewers independently extracted data from the remaining studies. The two datasets were compared, and final data were prepared for statistical analysis.

### 2.4. Statistical Analysis

Stata 13.0 was used to calculate the sensitivity (SN), specificity (SP), positive predictive value (PPV), negative predictive value (NPV), area under the curve (AUC), and receiver operating characteristic (ROC) curve. Forest plots comparing the SN and SP for each finding were developed. Revman 5.3 was used for meta-analysis.

## 3. Results

### 3.1. Literature Search and Article Selection 

**Figure 1:**
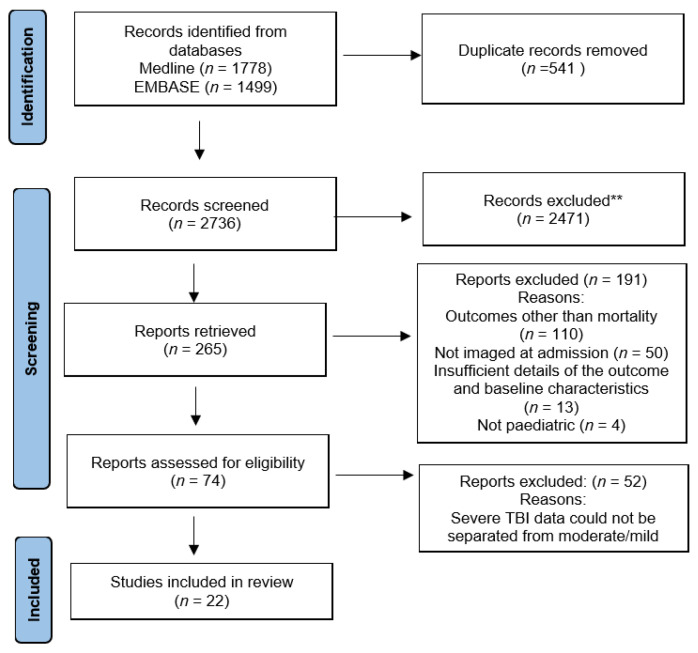
PRISMA flow chart. **2471 records were excluded because they met the exclusion criteria or did not include the inclusion criteria in Table 1.

Using the search criteria and after removing the duplicate records, a total of 2736 studies were included. On screening of these studies, 2471 studies did not meet the inclusion criteria for our systematic review. A total of 265 studies met the inclusion criteria for our systematic review. The reasons for exclusion of studies are listed in Figure 1. Another 52 studies had to be excluded as data of severe traumatic brain injury were mixed with that of mild and moderate traumatic brain injury and could not be separated. This left 22 studies that were included in the systematic review.

### 3.2. Summary of Demographics and Outcome

The final 22 studies ranged over 16 different countries including Canada, the United States, India, England, Australia, New Zealand, Israel, Austria, Switzerland, France, Italy, Russia, Germany, Malaysia, Argentina, and South Africa. All the studies were published between 1984 and 2019. The total number of patients was 2219 (Table 2). All the patients were ≤18 years old, had severe TBIs with a GCS less than 8, and were scanned with CT within 24 h. All the studies reported outcome with GOS. There was a combination of multi-center and single-center studies. All the studies included at least five patients. Table 2 shows the demographic data and imaging findings.

The number of total patients is 2219, but there are two discrepancies. Firstly, the number of males plus females is 2208 (discrepancy of 11). The discrepancy of the number of males plus females is because one study had a total sample of *n* = 16 with 8 males and 8 females [6]. However, only 11 of their patients had severe TBIs, so we only extracted data about imaging findings and outcomes of those 11 patients [6]. However, that study did not specify the sexes of those 11 patients. Secondly, the number of dead plus survived is 2015 (discrepancy of 4). The discrepancy in the dead plus survived is from another study that had a sample size of 77 but there were missing data on the outcome of four patients [8].

Furthermore, it is important to note that not every study reported on every imaging finding. Table 2 summarizes what was reported in all 22 studies, which may not represent the true proportion of the findings within the population. That is why the raw number and the percent are reported in the second column. Looking at the raw number could be misleading, because it could have been assumed that the prevalence of the finding is lower than what it truly is. The percentages may be more representative of the true proportion of clinical findings in patients presenting with severe TBI.

There was a total of 22 studies. Eleven studies reported SDH and 36.7% of all patients presented with SDH. Eleven studies also reported EDH and 8.8% of all patients presented with EDH. Fourteen studies reported tSAH and 30.0% of all patients presented with tSAH. Ten studies reported skull fractures and 31.6% of all patients had skull fractures. Ten studies also reported brain edema and 45.0% of all patients had brain edema. All 22 studies reported sex and outcome (dead or survived), as this was part of the inclusion and exclusion criteria.

### 3.3. Imaging Findings

**Figure 2:**
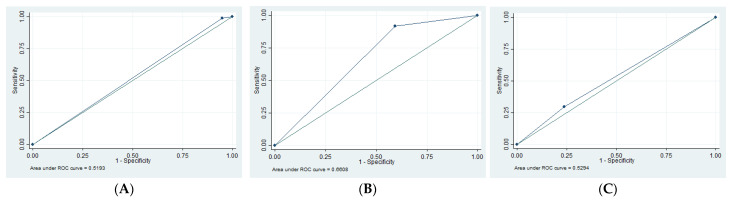

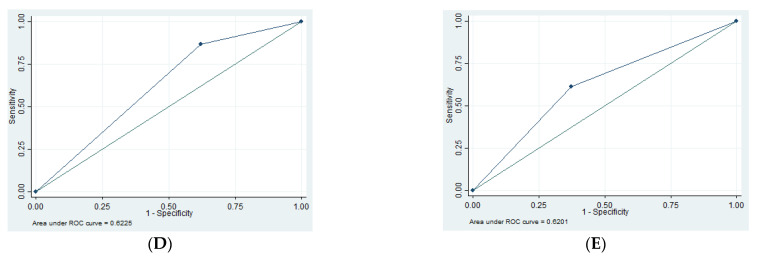
ROC for different parameters for diagnosis of death vs. survived. (**A**) ROC for EDH showing the AUC of 0.52. (**B**) ROC for edema showing the AUC of 0.66. (**C**) ROC for skull fractures showing the AUC of 0.53. (**D**) ROC for SDH showing the AUC of 0.62. (**E**) ROC for tSAH showing the AUC of 0.62.

**Figure 3:**
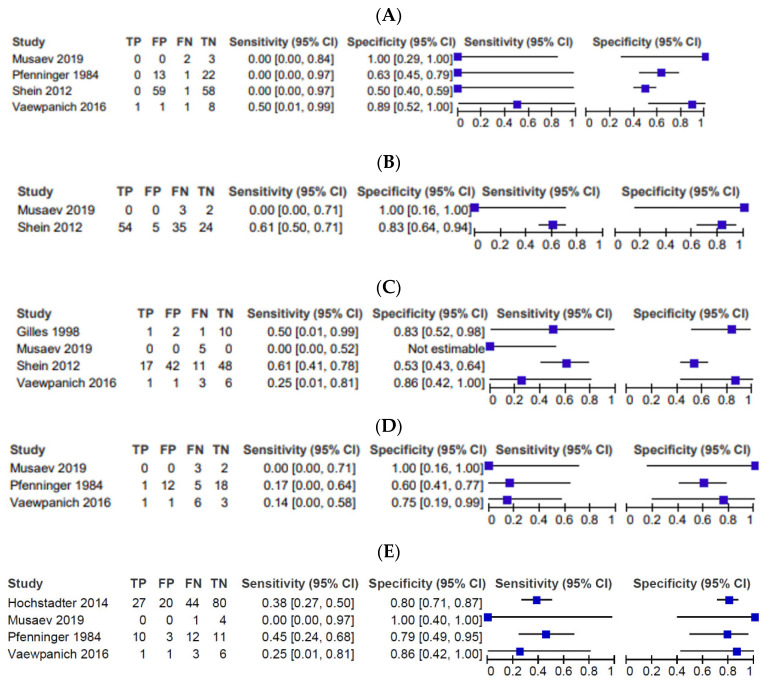
Forest plots for different imaging parameters for diagnostic accuracy of death vs. survival. (**A**) Forest plots showing the confidence interval for sensitivity and specificity of EDH. (**B**) Forest plots showing the confidence interval for sensitivity and specificity of edema. (**C**) Forest plots showing the confidence interval for sensitivity and specificity of skull fractures. (**D**) Forest plots showing the confidence interval for sensitivity and specificity of SDH. € Forest plots showing the confidence interval for sensitivity and specificity of tSAH.

In all 22 studies, 293 patients had SDH, 77 had EDH, 347 had tSAH, 244 had skull fractures, and 416 had cerebral edema (Table 2). Once again, not every study reported on every finding and some studies did not have patient-specific data relating each finding to a mortality rate (i.e., the rate of the finding was presented and the overall mortality rate was presented separately without insight on how many individuals with the finding ended up surviving). Nonetheless, this systematic review performed the meta-analysis based on the studies that did report outcome based on each imaging finding. Sensitivity (SN), specificity (SP), negative predictive value (NPV), positive predictive value (PPV), and area under the curve (AUC) in Table 3, the receiver operating characteristic (ROC) in Figure 2, and the forest plots in Figure 3 were calculated with the following: four studies for EDH [6,9,10,11], two studies for edema [10,11], four studies for skull fractures [6,10,11,12], three studies for SDH [6,9,11], and four studies for tSAH [3,6,9,11].

### 3.4. Risk of Bias

All included studies were retrospective studies with no control group or blinding of the readers, resulting in high bias and lower grade of evidence.

## 4. Discussion

Severe TBI is a serious clinical condition with over 25% mortality in our study. Clinical examination is limited in these patients. Imaging plays a significant role in initial assessment of these patients. We assessed if the findings on initial imaging could help predict mortality. This discussion incorporates a qualitative assessment from the majority of the 22 studies that fit the inclusion criteria. However, the quantitative analysis was only based on a few studies (referenced in the subsection titled Imaging Findings). Few studies were used for the quantitative assessments because patient-specific data for findings and outcomes were not available. Unfortunately, this is the disadvantage of a retrospective study, as we had no control over the existing data. We found that edema had the highest AUC (0.66). SDH and tSAH also had high AUCs (both 0.62). Skull fractures had an AUC of 0.53 and EDH had an AUC of 0.52.

### 4.1. Cerebral Edema

Cerebral edema on initial CT of the head had the highest diagnostic accuracy for the mortality in patients with severe TBI with an AUC of 0.66, SN of 92%, and NPV 84%. Many other studies also found that brain edema was a predictor of poor outcome [2,4,9,10,11,12,13]. In particular, Feickert et al. found that “edema was the most important factor contributing to poor outcome”, and they suggest that reducing edema after brain injury is pivotal to improving outcome [2]. Many studies emphasize the importance of controlling edema immediately in order to improve outcome [4,13]. However, one study did label edema as a predictor of “intermediate outcome” [14]. It is still unclear to what extent the cerebral edema seen on initial CT of the head is reversible.

### 4.2. SDH

SDH had a moderately high SP (62%) and NPV (64%) with an AUC of 0.62 for diagnosing mortality in severe TBI patients. Our findings show that 62% of people that survived did not have SDH and 64% of people without SDH survived. Thus, the lack of SDH on the initial CT is correlated with survival. A study commented that SDH had poor outcome [14]. This is consistent with our findings that SDH had a moderately high SP and NPV. However, one study said that chronic SDH had lower mortality, which is inconsistent with our findings [10].

### 4.3. EDH

Other studies noted that EDH was associated with good outcome [14,15]. Likewise, our study showed that EDH had an SP of 95%, indicating that 95% of people that survived did not have EDH. Similar to SDH, the lack of EDH on initial CT had good prognosis. Although EDH had the highest SP in our study, it had a low SN (1.35), PPV (16.67), NPV (55.49), and AUC (0.52).

### 4.4. tSAH

The literature had conflicting information on tSAH. Some studies found that tSAH was indicative of more severe TBIs but was not associated with outcome [3,16]. However, other studies did identify tSAH as an indicator of poor outcome [8,13]. Our results suggest that tSAH had a high NPV, meaning that 81% of patients without SAH survived. Therefore, the lack of tSAH on initial CT has good prognosis.

### 4.5. Skull Fractures

A couple of studies noted that head and skull fractures do not significantly influence outcome [2,8]. However, Shein et al. (2012) did cite a study that associated skull fractures with poor outcome. In our study, skull fractures had a relatively high SP, indicating that 76% survivors did not have skull fractures. Hence, the lack of skull fractures on initial CT has good prognosis.

### 4.6. Sex

Many studies noted that males were over-represented in their samples, but there was no significant difference in outcome between males and females [15,16,17]. However, the sex composition corresponding to each symptom was not reported in enough studies to perform a meta-analysis on these data. The over-representation of boys with TBIs is consistent with our understanding that boys tend to take more risks, and there is a social expectation that boys participate in more dangerous activities, therefore leading to a higher rate of injury [18].

### 4.7. Additional Findings

It is highly likely that many of these findings were present in an individual patient with severe TBI. Lack of patient-level data limited us from studying the combined effect of these CT findings on the final outcome.

Low GCS was consistently associated with poor outcome [10,19,20,21], and one study noted that all patients with GCS 3-4 died [15]. Age has a complicated relationship with outcome. Generally, children have better outcomes than adults [16]. Kan et al. (2009) suggests that children may have better outcomes than adults because developing brains have better neuroplasticity. Kan et al. (2009) also found that there was no significant correlation between age and outcome for children. However, Semple et al. (1998) found that children under 3 years old had worse outcomes than older children. Some studies noted that intraventricular hemorrhages (IVH) were associated with poor outcome [14], while other studies found that IVH was not associated with outcome [16]. Other indications of poor outcome include hypotension [17,19,22], hypoxia [17,19,22], cerebral autoregulation [23], severe diffuse brain injury [13], midline shift [8], intraparenchymal contusions [14], intraparenchymal hemorrhage [10], mass lesions [12,19], bilateral swelling [12], raised ICP for long periods [12], retinal hemorrhage [10], diabetes insipidus [9], injury severity score (ISS) [19], high blood glucose [17], and size of pupils [22]. Comorbidities contributing to poor outcome include post traumatic seizures [17] and post traumatic epilepsy [11]. Mode of injury can also be used for prognosis [15]. In Semple et al.’s (1998) study, children with abusive head trauma had worse outcomes than children with other severe TBIs.

Although non-contrast CT of the head is the most common modality used for imaging of a traumatic brain injury and is an excellent modality for imaging of anatomical structures, it is limited, as it does not give any functional information. Functional imaging of CT perfusion (CTP) is being proposed for imaging in patients with TBIs. A pilot study using CTP as initial imaging for severe TBI patients reported it can predict in-hospital mortality in these patients [24]. This is based on the principle that CTP helps determine the dead brain in cases of stroke [25,26]. The same principle can help us detect brain death in critically ill patients [27,28,29]. CTP has challenges of a variable protocol and the variable thresholds used for characterization of the brain perfusion [30]. With further evolution in the perfusion protocol, automated post-processing software, and with more experience, CTP is likely going to be more used for prognostication of patients with TBI. An ongoing prospective study on this topic will be critical to watch for [31]. Even with the success of this study, the limited use and experience of CTP in pediatric patients will remain challenging.

### 4.8. Limitations

There are some limitations to this systematic review. Firstly, most studies were retrospective in nature with no blinding or control trials. Therefore, all included studies had a high level of bias, resulting in only lower grade of evidence. Secondly, the calculations for SN, SP, PPV, NPV, ROC, and AUC (Table 3, Figure 2 and Figure 3) were only based on a few studies rather than all 22 studies. This is because many studies reported the prevalence of each finding but did not report data about how each finding was associated with outcome. Thus, we were only able to report the frequency of these data but were unable to perform bivariate analysis with these studies. Thirdly, several studies had different comparisons for age and outcome. Some studies compared the outcome of infants to the outcome of older children, while other studies had slightly different cutoff points. Although qualitative analysis was possible, quantitative analysis comparing the studies was not possible. Fourthly, many of the studies did not report on every finding. Thus, the prevalence of each finding presented in Table 2 is not comprehensive. Fifthly, we were not able to obtain detailed patient-level data, limiting us from reporting on the effect of combinations of findings on initial CT of the head. Finally, this study collected data from 1984 to 2019. CT imaging has changed substantially in this time, which may have affected the results.

## 5. Conclusions

Out of the different CT scan findings, brain edema had the highest SN, PPV, NPV, and AUC. EDH had the highest SP to predict in-hospital mortality. The presence of these features on initial imaging provide valuable information for prognostication of patients with TBIs. However, the level of evidence from our systematic review is low, and we do not expect a change in clinical practice based on our results. More studies are needed for a higher level of evidence before clinical management could be modified based on the initial imaging findings.

## Figures and Tables

**Table 1:** Inclusion and exclusion criteria.

Inclusion Criteria	Exclusion Criteria
patients should be imaged with CT scan within 24 h of admission the study must talk about prognostication outcomes the study contains clinical picture including Glasgow Comma Scale (GCS) the injury must be a severe TBI (GCS ≤ 8) the study must include discharge outcomes with Glasgow Outcome Scale (GOS) the study must contain patient demographics (age, sex) the study must contain at least 5 patients both single- and multi-center are acceptable	any studies with non-acute traumatic events any non-brain or non-calvarium injuries adult studies (over 18 years) any non-traumatic brain injury mild and moderate traumatic brain injuries all systematic reviews, meta-analyses, reviews, and book chapters

**Table 2:** Demographic data, prevalence of imaging findings, and outcome in all 22 studies.

Total Patients	2219	Number of Studies That Reported Finding
Male	1461	22
Female	747	22
Subdural hematoma	293/797 = 36.7%	11
Epidural hematoma	77/879 = 8.8%	11
Traumatic Subarachnoid hemorrhage	347/1154 = 30.0%	14
Skull fractures	244/771 = 31.6%	10
Brain edema	416/924 = 45.0%	10
Dead	564/2219 = 25.4%	22
Survived	1651/2219 = 74.4%	22

**Table 3:** Sensitivity (SN), specificity (SP), negative predictive value (NPV), positive predictive value (PPV), and area under the curve (AUC) for epidural hematoma (EDH), subdural hematoma (SDH), traumatic subarachnoid hemorrhage (tSAH), skull fractures (SF), and edema.

	SN	SP	PPV	NPV	AUC
**EDH**	1.35	94.79	16.67	55.49	0.52
**SDH**	13.3	62.16	12.50	63.89	0.62
**tSAH**	61.29	62.73	38.78	80.80	0.62
**SF**	29.69	76.19	48.72	58.72	0.53
**Edema**	91.53	40.63	58.70	83.87	0.66

## Data Availability

The study does not report any patient-level data, hence not applicable.

## Supplementary Materials

The following are available online at https://www.mdpi.com/article/10.3390/tomography9020044/s1. Word S1: Details of Search Strategy.

## Abbreviations

CT	computed tomography
TBIs	traumatic brain injuries
GCS	Glasgow Coma Scale
GOS	Glasgow Outcome Scale
SN	sensitivity
SP	specificity
PPV	positive predictive value
NPV	negative predictive value
AUC	area under curve
ROC	receiver operating characteristic
EDH	extradural hematoma
SDH	subdural hematoma
tSAH	traumatic subarachnoid hemorrhage
