# Supplementary material for: Initial CT Imaging Predicts Mortality in Severe Traumatic Brain Injuries in Pediatric Population—A Systematic Review and Meta-Analysis
Source: Tomography. 2023 Feb 27;9(2):541–51. doi: 10.3390/tomography9020044 (PMC10037655; doi:10.3390/tomography9020044)
Supplement: Supplementary file 1 [file tomography-09-00044-s001.zip › tomography-2188571-supplementary.pdf]

Database: Ovid MEDLINE(R) and Epub Ahead of Print, In-Process & Other Non-Indexed Citations and Daily <1946 to July 02, 2020>
Search Strategy:
----
1 Brain Injuries, Traumatic/ (6138)
2 Brain Injuries, Traumatic/di, dg [Diagnosis, Diagnostic Imaging] (1342)
3 brain injuries/ or brain hemorrhage, traumatic/ (52210)
4 Brain Injuries/dg [Diagnostic Imaging] (2766)
5 Brain Injuries/cl [Classification] (659)
6 Brain Injuries/mo [Mortality] (2059)
7 craniocerebral trauma/ or head injuries, closed/ or head injuries, penetrating/ or intracranial hemorrhage, traumatic/ or exp brain hemorrhage, traumatic/ or subarachnoid hemorrhage, traumatic/ (27081)
8 hematoma, subdural/ or hematoma, subdural, acute/ or hematoma, subdural, intracranial/ (7695)
9 (traumatic brain or traumatic head or severe brain or severe head).ti. (24995)
10 (2 or 3 or 4 or 5 or 6) and traumatic.ti,ab,sh. (22474)
11 (2 or 3 or 4 or 5 or 6) and severe.ti,ab,sh. (10414)
12 10 or 11 (26306)
13 (severe or traumatic).ti,ab,sh. (1069969)
14 8 and 13 (1623)
15 (acute or hyperacute).ti,ab,sh. (1178752)
16 or/1-7 (81485)
17 (15 and 14) or (15 and 16) (9250)
18 17 and (10 or 11) (4128)
19 1 or 7 or 9 or 12 or 14 or 18 (62655)
20 tomography, emission-computed/ or positron-emission tomography/ or positron emission tomography computed tomography/ or tomography, emission-computed, single-photon/ or single photon emission computed tomography computed tomography/ or tomography, x-ray computed/ or computed tomography angiography/ or exp tomography, spiral computed/ (487065)
21 exp Magnetic Resonance Imaging/ (451132)
22 exp Magnetic Resonance Spectroscopy/ (209544)
23 (magnetization transfer imaging or magnetic source imaging).mp. (806)
24 Neuroimag*.ti,ab,sh. (55222)
25 ultrasonography/ or echoencephalography/ or ultrasonography, doppler, transcranial/ or ultrasonography, doppler/ or diagnostic techniques, neurological/ or exp neuroimaging/ (365826)
26 20 or 21 or 22 or 23 or 24 or 25 (1321145)
27 (predict* or prognos* or detect* or longitudinal* or long term or projection* or projecting or forecast* or foretell* or morbid* or mortalit* or surviv* or death* or scoring system* or Glasgow or rotterdam or modified rankin or marshall or Helsinki or full outline or four scale or classif* or sequela*).ti,ab,sh. (7375057)
28 trauma severity indexes/ or injury severity score/ (24113)
29 27 or 28 (7383569)
30 19 and 26 and 29 (5360)
31 (mild or minor or concussion* or postconcussion*).ti. (60633)
32 30 not 31 (4525)
33 (severe and mild).ti. (1312)
34 30 and 33 (5)
35 32 or 34 (4530)
36 (comment or editorial or news or newspaper article or case reports).pt. (3517101)
37 (letter not (letter and randomized controlled trial)).pt. (1081760)
38 36 or 37 (3950246)
39 35 not 38 (3697)
40 limit 39 to english language (3352)
41 exp child/ or exp "congenital, hereditary, and neonatal diseases and abnormalities"/ or exp infant/ or adolescent/ or exp pediatrics/ or child, abandoned/ or exp child, exceptional/ or child,

orphaned/ or child, unwanted/ or minors/ or (pediatric* or paediatric* or child* or newborn* or congenital* or infan* or baby or babies or neonat* or pre-term or preterm* or premature birth* or NICU or preschool* or pre-school* or kindergarten* or kindergarden* or elementary school* or nursery school* or (day care* not adult*) or schoolchild* or toddler* or boy or boys or girl* or middle school* or pubescen* or juvenile* or teen* or youth* or high school* or adolesc* or pre-pubesc* or prepubesc*).mp. or (child* or adolesc* or pediat* or paediat*).jn. (5071974)
42 40 and 41 (1778)

****

Filter used:
Tjosvold, Lisa, Sandy Campbell, Marlene Dorgan. Filter to Retrieve Pediatric Articles in the OVIDMedline Database. John W. Scott Health Sciences Library, University of Alberta. Rev. April 18, 2020
https://guides.library.ualberta.ca/c.php?g=342568&p=5096194

Database: Embase <1974 to 2020 Week 26>
Search Strategy:
----
1 traumatic brain injury/ or pediatric traumatic brain injury/ (49085)
2 (traumatic brain or traumatic head or severe brain or severe head or head trauma or tbi).ti. (40690)
3 1 or 2 (63094)
4 (mild or minor or concussion* or postconcussion*).ti. (77298)
5 3 not 4 (56508)
6 (severe and mild).ti. (1790)
7 3 and 6 (141)
8 5 or 7 (56649)
9 computer assisted diagnosis/ or computer assisted radiography/ or neuronavigation/ (44654)
10 computer assisted emission tomography/ or exp computer assisted tomography/ or emission tomography/ (1060309)
11 nuclear magnetic resonance imaging/ or diffusion tensor imaging/ or diffusion weighted imaging/ or dynamic contrast-enhanced magnetic resonance imaging/ or echo planar imaging/ or fluorine magnetic resonance imaging/ or functional magnetic resonance imaging/ or interventional magnetic resonance imaging/ or magnetic resonance venography/ or multiparametric magnetic resonance imaging/ or perfusion weighted imaging/ or susceptibility weighted imaging/ or whole body mri/ (896234)
12 nuclear magnetic resonance spectroscopy/ (116713)
13 exp neuroimaging/ (137083)
14 neuroimag*.ti. (12572)
15 echography/ or contrast-enhanced ultrasound/ or doppler ultrasonography/ or echoencephalography/ or echotomography/ or exp focused assessment with sonography for trauma/ or high frequency ultrasound/ or interventional ultrasonography/ or intravascular ultrasound/ or radiofrequency echographic multi spectrometry/ or real time echography/ or three dimensional echography/ (349950)
16 exp brain radiography/ (52962)
17 or/9-16 (2166652)
18 (predict* or prognos* or detect* or longitudinal* or long term or projection* or projecting or forecast* or foretell* or morbid* or mortalit* or surviv* or death* or scoring system* or Glasgow or rotterdam or modified rankin or marshall or Helsinki or full outline or four scale or classif* or sequela*).ti,ab,hw. (10466633)
19 injury scale/ (20984)

20 18 or 19 (10472203)
21 8 and 17 and 20 (6886)
22 21 not ((exp animal/ or exp invertebrate/ or nonhuman/ or animal experiment/ or animal tissue/ or animal model/ or exp plant/ or exp fungus/) not (exp human/ or human tissue/)) (6461)
23 limit 22 to (article or article in press or "review") (4511)
24 case report*.ti,kw. (314730)
25 22 and 24 (162)
26 23 not 25 (4390)
27 limit 26 to english language (4177)
28 juvenile/ or exp adolescent/ or exp child/ or exp postnatal development/ or (pediatric* or paediatric* or child* or newborn* or congenital* or infan* or baby or babies or neonat* or pre term or preterm* or premature birth or NICU or preschool* or pre school* or kindergarten* or elementary school* or nursery school* or schoolchild* or toddler* or boy or boys or girl* or middle school* or pubescen* or juvenile* or teen* or youth* or high school* or adolesc* or prepubesc* or pre pubesc*).mp. or (child* or adolesc* or pediat* or paediat*).jn. (4681140)
29 27 and 28 (1499)

****

Filter used:
Desmeules, Robin. Filter to Retrieve Pediatric Articles in the OVID EMBASE Database. John W. Scott Health Sciences Library, University of Alberta. Rev. April 18, 2020
https://guides.library.ualberta.ca/c.php?g=342568&p=5096194
